# Attitudes toward artificial intelligence and its application in psychotherapy: Assessment in healthy adults and validation of an assessment measure

**DOI:** 10.1177/20552076251410978

**Published:** 2026-03-12

**Authors:** Jannis Nagel, Karsten Hollmann, Annika K Alt, Tobias J Renner, Annette Conzelmann

**Affiliations:** 1 Department of Psychology (Clinical Psychology II), 227872 PFH – Private University of Applied Sciences, Göttingen, Germany; 2 Department of Child and Adolescent Psychiatry, Psychosomatics and Psychotherapy, 88861 University Hospital of Psychiatry and Psychotherapy, Tübingen, Germany; 3 German Center for Mental Health (DZPG), Partner Site, Tübingen, Germany

**Keywords:** Artificial intelligence, attitudes, psychotherapy, interindividual differences, personality, mental health

## Abstract

**Objectives:**

Artificial intelligence (AI) represents a digital turning point that might have an impact on psychological treatment. To effectively integrate AI into psychological practice, it is important to analyze attitudes toward AI and the factors influencing these attitudes.

**Methods:**

To achieve this objective, a quantitative online survey to assess acceptance of AI was designed and assessed in 205 adult participants. Additionally, demographic variables, psychological symptoms, psychotherapy experience, and personality traits were assessed as potential factors influencing AI acceptance.

**Results:**

In general, attitudes toward AI and its application in psychotherapy were relatively neutral. The results indicated that men, academics, and people without psychological symptom burdens showed lower anxiety of AI in general. Conscientiousness and extraversion correlated negatively with the acceptance of AI in general. Concerning the application of AI in psychotherapy, the only significant difference was found that men showed a more positive attitude compared to women. The most accepted areas were the application of AI in diagnostics and app-based interventions, the lowest acceptance was found for the application of robotics.

**Conclusions:**

The main finding emphasizes that AI in general and in relation to its application in psychotherapy is considered as neutral and can be used in diagnostic assessments and treatment, although the type of AI might be important. For some subgroups of patients, it might be important to increase their acceptability concerning the application of AI within psychotherapy if this is intended to be embedded in the therapeutic process.

## Introduction

Artificial intelligence (AI) with technical or digital tools based on algorithms or deep learning has become ubiquitous in recent years and has an impact on various aspects of modern life. The range of applications of AI is broad, and it may also prove valuable in psychotherapeutic domains by creating more accessible treatment options and reducing costs.^1,2^ Research currently shows effective applications in the treatment of patients with a wide range of disorders.^3–7^ This is particularly relevant given the limited availability of psychotherapeutic experts. In contrast, internet access or smartphones are widely available.^8^ AI can be applied across the psychotherapeutic process, including diagnostics, monitoring, and treatment, and is expected to develop rapidly in the future.^9,10^

Our study focuses on attitudes of potential users of AI in the diagnostic and treatment process of mental disorders. Diagnostic tools include AI applications based on deep learning, algorithms or wearables, such as web-based symptom checkers or decision support systems for mental disorders.^11,12^ AI offers innovative methods to enrich diagnostic processes by analyzing biological markers, acoustic, visual, and textual features, instead of relying solely on clinical interviews and self-assessments. These advances are poised to improve the accuracy and efficiency of diagnostic processes, they can help to identify, specify or predict symptoms.^9,10^

Beyond diagnostic applications, AI is increasingly being used to support core areas of the psychotherapeutic process itself. It helps to monitor it, to give timely feedback, to send reminders and to provide information about the patient's status.^9,13^ AI in the form of algorithms or natural language processing via chatbots or within mental health apps supports engagement, adherence, constant availability, self-expression, emotional processing, treatment outcomes themselves and allows to overcome barriers to treatment.^9,14–20^ Embodied AI in the form of socially interactive robots opens up innovative possibilities for enhancement of well-being, skill trainings, promotion of personal autonomy, therapeutic engagement and motivation, while at the same time raising important ethical questions.^15,16,21^

While general attitudes toward AI have been increasingly studied, acceptance of AI specifically within psychotherapy constitutes a distinct and more sensitive domain. This distinction is crucial because therapeutic settings rely heavily on trust, emotional safety, and human interaction, which may shape attitudes differently than general AI applications. Despite numerous arguments in favor of its application, attitudes toward AI in psychotherapy are still largely unexplored. Nevertheless, acceptance and a positive attitude toward AI could be essential for its integration into diagnostics and therapeutic interventions and treatment success.

Attitudes toward AI seem to be heterogeneous, especially regarding its application in healthcare.^22–26^ A meta-analysis revealed that the acceptance of AI can be divided into three different domains that include factors related to people, the AI systems themselves (e.g. area of application), and the context in which their interactions take place.^22^ One finding from this and another study is that personality traits might have a significant impact on the trust people place in AI.^24^ Younger age, higher education levels and male gender could be associated with a higher acceptance of AI.^27^ Attitudes toward the application of AI for clinical diagnoses differ between well-informed AI experts and the general population. Over 90% of AI experts consider AI to be a valuable tool for clinical diagnoses, while the approval rate in the general population is just over 50%.^28^ Accordingly, further research is required to attitudes toward AI in the mental health section and individual factors influencing these attitudes.

Especially important for attitude toward AI are also its area of application.^22,29^ The approval rate for internet-based interventions is up to 81%.^30^ The application of AI in psychotherapy is viewed more skeptically and should be supplemented by human supervision.^26,31^ Criticism from both professionals and patients relates to the perceived inadequacy of AI in acquiring skills such as emotional intelligence, empathy and the ability to actively listen, which enhance the therapeutic relationship and treatment outcomes.^32^ The characteristics of AI, such as unpredictability, changeability could increase patients’ anxiety.^33^ Nevertheless, attitudes toward the integration of AI within psychotherapeutic settings are still a largely unexplored area.

This study addresses a critical gap in the literature by directly assessing attitudes toward AI specifically within the context of psychotherapy, rather than focusing on general healthcare, other technical contexts or mental health allocations. A contribution to the understanding of acceptance of AI in a domain where human interaction and trust are particularly essential is required. The application of AI into the psychotherapeutic process depends on acceptance from its users. The general population's attitudes toward the use of AI in psychotherapy are still unclear as well as contextual and individual factors that might have an influence on these attitudes. The aim of this study was, therefore, to assess the attitudes of the general population toward AI tools in general and in relation to diagnostics and psychotherapy and to investigate the influence of personality dimensions, socio-demographic variables, psychopathology, and different areas of application of AI within the psychotherapeutic process (e.g. diagnostics, apps, chatbots, robots). Notably, there is a lack of validated instruments that capture nuanced attitudes toward AI-driven interventions in psychotherapy. Existing AI attitude measures primarily assess general or technology-focused perceptions and do not capture the specific therapeutic dimensions relevant to psychotherapy. Therefore, a new domain-specific instrument was required to adequately assess attitudes toward AI within psychotherapeutic contexts. Accordingly, we developed the scale Attitudes Toward the Application of Artificial Intelligence in Psychotherapy (ATUAIP), a purpose-built instrument that captures attitudes toward AI within psychotherapy more comprehensively than existing measures. Our results might offer practical implications for the responsible implementation of AI in psychotherapy. We expected to identify more positive attitudes toward AI among males, people with younger age, higher academic education, less psychological symptom burden and higher openness and extraversion and areas of applications with the guidance of AI by humans rather than autonomic processes.

## Research method

### Sample

Sample size was planned a priori using G*Power 3.1 with *α* = 0.05 (two-tailed) and power of 1 - *β* = 0.80. Power analyses were performed for independent-sample *t*-tests (equal group sizes), correlations, and multiple regressions. For group comparisons, the required sample size was *N* ≈ 64 per group (*N* ≈ 128 in total) for an expected effect of *d* = .50. For correlation analyses, *N* ≈ 193 cases were required for *r* = 0.20. For multiple regression analyses with up to 15 predictors, the required sample size was *N* ≈ 139 (*f*^2^ = .15). In addition, a stable sample size was desirable for the scale development of the ATUAIP. Previous methodological work has recommended a minimum sample size of approximately *n* ≥ 200 to ensure robust factor solutions.^34^ To account for potential data loss due to exclusions or incomplete responses and to buffer the reduction in power resulting from multiple testing, the recruitment target was set at ≥200. A total of 205 participants were finally included. Incomplete responses were removed before analysis, and missing values in the final dataset were managed via listwise deletion. Participants were recruited through a range of channels, including university online platforms, social media advertising and offline methods such as print advertisements.

### Instruments

#### BFI (Big Five Inventory)

The BFI-10 is a short version of the Big Five Inventory, designed to assess the five major dimensions of personality: Openness, conscientiousness, extraversion, agreeableness, and neuroticism.^35^ This 10-item inventory is widely used due to its brevity and reliability and was selected because it allows for an efficient and validated assessment of the core five personality dimensions in a very brief format, making it particularly suitable for survey-based research with limited participant time. Each dimension is represented by two items, scored on a 5-point Likert scale ranging from 1 (strongly disagree) to 5 (strongly agree). A study reported retest reliability coefficients (rtt = 0.49–0.84) over 6 weeks (*N* = 184) and a mean principal scale correlation of *r* = 0.69 with the NEO-PI-R, indicating good construct validity.^36^ A validation study from 2023 (*N* = 60,000) confirmed the structural stability and satisfactory factorial validity of the BFI-10, with internal consistency estimates of *α* = 0.44 and *Ω* = 0.53, replicating previous goodness-of-fit criteria.^37^

#### ICD-10-Symptom-Rating (ISR)

The ISR is an instrument for measuring the current level of psychological distress based on the symptoms associated with the disorders outlined in Chapter F of the ICD-10 classification.^38^ It includes six scales (depression, anxiety, somatoform symptoms, obsessive-compulsive symptoms, eating disorders, and additional measures). The ISR was selected because it is a well-validated and reliable self-report instrument that enables efficient assessment of many of the most common psychological disorders. Participants rate their experience of various symptoms over the past week on a 5-point Likert scale, ranging from 0 (not at all) to 4 (extremely). The German version of the ISR demonstrated high reliability, with a Cronbach's alpha of *α* = 0.92 for the overall score and *α* = 0.78 to 0.86 for individual syndrome scales.^39^ The test-retest reliability ranged from *r* = 0.82 to 0.89 for the total scale and *r* = 0.70 to 0.94 for individual scales across clinical and non-clinical samples, depending on the retest interval.^40^ External validation using DIA-X diagnoses confirmed the validity of the syndrome scales, with factor analysis resolving 73% of the variance in over 12,000 subjects.^39^

#### Attitude toward artificial intelligence (ATAI)

The ATAI measures individuals’ attitudes toward AI. This instrument assesses various aspects of attitudes, including perceived usefulness, trust, and ethical concerns regarding AI using two scales: Acceptance of Artificial Intelligence (ATAI_A) and Fear of Artificial Intelligence (ATAI_F) and was therefore very well suited to investigating the research questions of this thesis. The two scales are measured with a total of five items, three of which were assigned to the fear scale and two to the acceptance scale, which were asked in this study on a five-point Likert scale ranging from 1 (“doesn’t apply at all”), 2 (“doesn’t really apply”), 3 (“neither nor”), 4 (“is more likely to apply”), to 5 (“does completely apply”). The ATAI has been developed to capture the complex and multifaceted nature of attitudes toward AI, ensuring comprehensive assessment.^41^ Internal consistencies were *α* = 0.65 for the acceptance scale and *α* = 0.66 for the anxiety scale, which is considered acceptable given the small number of items. Additionally, the acceptance scale correlated positively with willingness to use AI products, while the anxiety scale showed negative correlations.^41^

#### Attitude toward the application of artificial intelligence in psychotherapy (ATUAIP)

Previous findings on the application of AI indicate that attitudes in medicine, healthcare, and psychology are complex, subjective, and context dependent. Since there are currently no publications on attitudes toward AI in psychotherapy, it was necessary to create new items and to develop and valid scale. For this purpose, a pool of 10 items was created to cover various aspects of AI in different areas of application and to represent a scale (attitudes toward the use of AI in psychotherapy). The items can be found in Table 1. The German version is available from the authors. The item formulations were based on theoretical assumptions. The areas in which AI is already used in psychotherapy were analyzed. Some of the most widespread areas of application were selected. Participants were instructed to answer these items using a five-point Likert scale, ranging from 1 (“strongly disagree”) to 5 (“strongly agree”), 3 = neither nor. There were questions on the application of AI in the diagnostic and therapeutic process and on the use of chatbots, robots or apps in specific.

**Table 1.** Items of the attitude toward the use of artificial intelligence in psychotherapy (ATUAIP).

I could imagine that it would make sense to use artificial intelligence in psychological conversations. I could imagine AI-based programs helping me structure my day. I could imagine conducting therapy sessions with an artificial intelligence if these are regularly reviewed (e.g. once a month) with psychologists. I could imagine conducting diagnostic interviews with an artificial intelligence if the therapy is subsequently conducted with psychologists. I could imagine conducting psychotherapy exclusively with an artificial intelligence. I could imagine conducting a psychotherapy session solely with an artificial intelligence to bridge the waiting time for a therapy slot. I could imagine building a therapeutic relationship with an artificial intelligence. The use of chatbots would be useful in the context of psychotherapy (a chatpot is intelligent software that allows users to chat). The use of robots with human facial features would be useful in the context of psychotherapy. The use of app-based support would be useful in the context of psychotherapy.

*Note*. Items rage from 1 (“doesn’t apply at all”), 2 (“doesn’t really apply”), 3 (“neither nor”), 4 (“is more likely to apply”), 5 (“does completely apply”).

In order to examine the attitudes of the participants in a differentiated manner, various possible applications of AI were identified and surveyed. To validate the generated item pool and consolidate it into a scale, both exploratory and confirmatory factor analyses were conducted. Both the exploratory and confirmatory factor analyses were conducted on the full sample. The Kaiser–Meyer–Olkin criterion (KMO) was used to assess the feasibility of exploratory factor analysis (EFA) based on the available items. The KMO coefficient was 0.89, indicating that the data were suitable for factor analysis. Additionally, Bartlett's test yielded a significant result (*p* < 0.001), further confirming the feasibility of the EFA. The results of the EFA show that all items loaded onto a single factor, even though a second subscale with two items would have been possible.

The results and the intercorrelation matrix resulting from the factor analysis can be obtained from the authors on request. After removing two items during the EFA, the final ATUAIP scale comprised eight items. The subsequent confirmatory factor analysis (CFA) showed a good model fit. The CFA yielded the following ratios: comparative fit index (CFI = 0.973), Tucker–Lewis index (TLI = 0.962), root mean square error of approximation (RMSEA = 0.059), and standardized root mean square residual (SRMR = 0.038). Furthermore, the scale had an internal consistency of *α* = 0.85. Additionally, cross-validation with the ATAI scale revealed a positive correlation with acceptance of AI (*ρ* = 0.45) and a negative correlation with anxiety about AI (*ρ* = -0.38).

### Procedure

A quantitative cross-sectional survey was conducted in German. The data were collected online via LimeSurvey^42^ over 2 months in the year 2023. Participants were recruited through various channels, including university online platforms, social media, and print media. Inclusion criteria were a minimum age of 18 years, German language proficiency, and internet access. A raffle was offered as an incentive. All participants gave informed consent and agreed to the data protection guidelines. The study was approved by the ethics committee of the PFH faculty.

The survey was divided into several sections; at the beginning, participants were informed about the definitions of AI and psychotherapy. Afterwards, they were asked to provide information about demographic characteristics, psychotherapeutic treatment experience, age, gender, and current level of education. Subsequently, participants completed surveys related to the Big Five personality structure using the BFI-10, on their current psychological symptom burden using the ISR, on attitudes toward AI with the ATAI and on their attitudes toward the application of AI in psychotherapy with the ATUAIP.

### Statistical analysis

The statistical analysis was performed using R Studio. A factor analysis was calculated for the self-developed ATUAIP.

The participants were then divided into different groups. This involved categorizing participants based on the ISR disorder syndromes. Cut-off values for at least a low symptom burden according to the IQP (2010) were used for this categorization. Six new scales with the values 0 (“no symptom burden”) and 1 (“at least low symptom burden”) were created for people who indicated at least a low symptom burden on the five disorder-related ISR scales and the overall scale. Additionally, for education, an academic group consisting of participants who reported a university degree or a doctorate as their highest educational qualification was classified as compared to non-academics.

After classification, the data were examined to ensure the requirements for the analysis procedures were met. For this purpose, the normal distribution was checked using the Shapiro–Wilk test. As the Shapiro–Wilk tests showed that several variables violated normality assumptions, non-parametric Wilcoxon rank-sum tests were used. Graphical tests for outliers, homoscedasticity and linear correlation were performed.

Next, the group differences in terms of gender, educational level, therapy experience and the previously classified groups were examined in relation to the anxiety scale and acceptance scale of the ATAI and the ATUAIP scale. For variables that did not meet with the normal distribution assumption, the Mann–Whitney *U* test (also Wilcoxon rank sum test), a non-parametric alternative to the *T*-test, was utilized.

To investigate the relationships between the metric variables and the AI scales, bivariate correlations between age, BFI-subscales, ISR-subscales and the AI-scales were calculated using the Spearman correlation. Subsequently, the correlations of the BFI-scales and the ISR-scales with the three AI-related scales were tested in multiple regressions. An *α*-level of 0.05 was used for all statistical tests.

## Results

### Sample descriptives

The sample consisted of 205 participants aged 18 to 85 years (*M* = 36.58, *SD* =17.48). 56.59% of participants were female, 40.98% male, and 2.43% diverse or unspecified. Educational backgrounds ranged with 57.56% from school degrees (“high school diploma”, “secondary school”, “comprehensive school”) or completed apprenticeships to academic background (bachelor, master or doctorate; 40.49%). Four participants did not provide any information on their educational background. Seventy-seven participants had current or previous psychotherapeutic experience. In terms of psychopathological characteristics, the respondents showed an above-average symptom burden overall. 48.7% of individuals displayed at least a low symptom burden indicating depressive symptoms, 42.9% symptoms related to anxiety disorders, 39.5% symptoms relating to obsessive-compulsive disorders (OCD), 21.5% somatoform symptoms, 32.6% symptoms associated with eating disorders, and 43.4% overall burden of psychological symptoms. The mean and SDs for the ICD-10 symptom rating and the Big Five Inventory were: depression (*M* = 0.93, *SD* = 0.75), anxiety (*M* = 0.95, *SD* = 0.87), somatoform symptoms (*M* = 0.46, *SD* = 0.75), obsessive-compulsive symptoms (*M* = 0.72, *SD* = 0.79), eating disorders (*M* = 0.72, *SD* = 0.86), openness (*M* = 3.52, *SD* = 0.95), conscientiousness (*M* = 3.60, *SD* = 0.84), extraversion (*M* = 3.49, *SD* = 0.95), agreeableness (*M* = 3.22, *SD* = 0.78), and neuroticism (*M* = 3.00, *SD* = 0.93).

### Analyses of ATAI and the ATUAIP

Overall, participants were indifferent regarding acceptance (*M* = 3.11, *SD* = 0.73) and fear of AI (*M* = 2.87, *SD* = 0.81) in general and its application in psychotherapy (*M* = 2.82, *SD* = 0.85). To assess the relationship between acceptance of AI in general and attitudes toward its application in psychotherapy, a paired Wilcoxon rank sum test revealed a better attitude toward AI in general (*V* = 13278, *p* < 0.001, *r* = 0.338).

Regarding the different areas of application in psychotherapy, the results show that the highest acceptance was reported for the use of app-based interventions (*M* = 3.3, *SD* = 1.18) and diagnostics (*M* = 3.23, *SD* = 1.20), followed by general use (*M* = 2.8, *SD* = 1.17) and chatbots (*M* = 2.72, *SD* = 1.20), while the lowest acceptance was observed for the use of robots (*M* = 2.29, *SD* = 1.19). A Wilcoxon rank-sum test showed significant differences for almost all listed constellations (*p*s < 0.001, *r*s > 0.41). No significant difference was found between the use of app-based interventions and the use in diagnostics.

In Supplement 1, we report further descriptives, analyses and effect sizes. Table S1 provides information on group differences regarding the AI-scales separately presented for gender, education and therapy experience, Table S2 on group differences regarding the AI-scales by symptom burden in the ISR-scales, Table S3 on differences in attitudes depending on the application areas and Table S4 on bivariate correlations of the AI-scales with the BFI and age. Gender: Analyses revealed that women had a significantly less positive attitude toward AI in general and its use within psychotherapy (all *p*s < 0.007, *r*s > 0.19). Education and mental symptom burden: In the group with low level of education and in those with a higher symptom burden (with the exception of eating disorders), the participants showed an increase in general anxiety of AI (*p*s < 0.041, *r*s > 0.14). There were no significant results for AI acceptance in general or its use within psychotherapy.

### Therapy experience

There were no significant effects on attitude toward AI in general or its use within psychotherapy. Concerning bivariate correlations between the AI measures, BFI, ISR and age, the only significant effects were negative correlations between acceptance of AI in general and extraversion and conscientiousness of the participants (*p*s < 0.05, *r*s > 0.10).

## Discussion

The aim of this study was to assess attitudes toward AI in general and its application in psychotherapy, as well as to identify influencing factors. Overall, attitudes were neutral, with slightly more positive views toward AI in general. These neutral ratings may reflect a combination of cautious openness and uncertainty due to limited personal experience with AI-based psychotherapeutic tools. Nevertheless, this neutrality might also encourage clinicians to include AI where it is helpful as its application is not principally rejected by the users. Acceptance was higher for AI in diagnostic procedures and app-based applications, whereas the use of robots in psychotherapy received lower approval. The corresponding effect sizes marked medium to strong effects. Academics reported lower fear of AI in general, but there was no significant correlation for the acceptance of AI or attitudes toward its application in psychotherapy. Men showed significantly lower anxiety and higher acceptance of AI in general and its application in psychotherapy. However, the corresponding effect sizes were small. Age had no effect. Regarding the influence of the Big Five personality facets on attitudes toward AI, two significant weak negative correlations were identified between conscientiousness and extraversion with the acceptance scale of AI in general. Individuals burdened by symptoms of a mental disorder showed a greater fear of AI in general. However, no significant differences were found in the scales measuring the acceptance of AI and its application in psychotherapy. No significant influence of therapy experience on attitudes toward AI was found. Overall, the perceived area and modality of AI application exerted a substantially greater influence on attitudes than individual demographic or personality factors.

Some of these findings are consistent with the few other studies regarding attitudes toward AI, such as effects of gender, academic status and psychological symptom burden.^26,27^ Other findings require further explanation. In contrast to other reports,^27^ we did not find an effect of age. Given the high level of education and relatively young age distribution of our participants, it is conceivable that the interaction of age and attitudes toward AI is modulated by the education variable.^28,43^ Personal factors are likely to explain attitudes toward AI significantly less than considerations regarding the area of application and the type of application.^22,30^ The less positive attitudes toward the use of robotics may be explained by greater familiarity with apps and app-based interventions compared to chatbots and robots.^29,44^ Neither neuroticism nor the ISR anxiety scale showed a significant correlation with fear of AI in general. While this may tentatively suggest that AI-related anxiety could be independent of general anxiety, such an interpretation should be treated with caution and requires replication in future research.

Beyond these methodological considerations, it is also important to reflect on what the present findings might imply for clinical practice and the broader use of AI in psychotherapy. The findings may have several practical implications. In line with other reports,^26^ this study found that participants were most open to diagnostic and app-based interventions, while robot-assisted applications were met with the greatest skepticism. Consistent with meta-analytic findings,^22^ this underlines the importance of transparency and user-centered design to build trust in AI tools and to foster acceptance, particularly in more vulnerable subgroups. In accordance with other studies on ethical considerations,^21^ the results also highlight the need for clear regulatory frameworks that address concerns such as reliability, empathy, and data security when implementing AI in psychotherapy.

## Limitations

Several limitations of this study should be mentioned. Assessing symptom burden from self-reports is not equivalent to verified clinical diagnoses by experts. For conclusions about the relationship between attitudes toward AI and clinical symptoms, future studies should consider the assessment of verified symptoms by clinical experts and also symptom burden. Assessing patients in psychotherapy would also be interesting. Further studies could also consider larger sample sizes and more heterogeneous samples to assess influencing attitudes toward AI, including different cultural groups.^24^ Our sample was skewed toward younger and more educated participants. Attitudes toward AI in psychotherapy might be different in less educated or older people. Additionally, the observed effect sizes were mostly small, limiting the explanatory power of the identified relationships. Future studies may benefit from broader recruitment strategies, including social media, paper-pencil questionnaires, and outreach in community settings (supermarket pinboards, community events). One other limitation of the present study is that the exact proportions of participants recruited via university platforms, social media, and offline channels were not systematically recorded; as such, recruitment bias related to channel distribution cannot be conclusively assessed. This should be assessed in future studies. Data were collected exclusively in Germany and therefore reflect the German healthcare system. Finally, a major limitation is the low internal consistency of the ATAI acceptance subscale, which restricts the interpretability of the findings. Moreover, the ATUAIP, although showing good psychometric properties in this study, requires independent validation in future research before it can be considered a robust measure.

## Conclusion

In summary, the present study has provided insights into attitudes toward AI and its potential application in psychotherapy, considering individual, demographic, and situational factors. Overall, people seemed to be open to AI and its application in psychotherapy and probably, AI should be cautiously included as one further possibility to provide or enhance diagnostics and psychotherapy and access to them. Efforts should be made to increase acceptance in certain subgroups of people. These groups might benefit from more information and instruction to the usefulness and possible applications of AI in general and within psychotherapy. Human support during the use of AI might be important. It may be important to increase the trustworthiness of applications such as robots or other AI tools with reduced human guidance when they are used in psychotherapeutic settings. The ATUAIP may further support clinical and educational practice by helping therapists to assess patients’ acceptance of AI-based tools. The possibilities by new technologies are developing rapidly and attitudes toward them and usability should be assessed continuously.^10,21,45–47^ In this context, long-term studies, follow-up investigations or cohort analyses offer interesting research perspectives.

## Supplemental Material

sj-docx-1-dhj-10.1177_20552076251410978 - Supplemental material for Attitudes toward artificial intelligence and its application in psychotherapy: Assessment in healthy adults and validation of an assessment measure Supplemental material, sj-docx-1-dhj-10.1177_20552076251410978 for Attitudes toward artificial intelligence and its application in psychotherapy: Assessment in healthy adults and validation of an assessment measure by Jannis Nagel, Karsten Hollmann, Annika K Alt, Tobias J Renner and Annette Conzelmann in DIGITAL HEALTH
