# Supplementary material for: Attitudes toward artificial intelligence and its application in psychotherapy: Assessment in healthy adults and validation of an assessment measure
Source: Digit Health. 2026 Mar 12;12:20552076251410978. doi: 10.1177/20552076251410978 (PMC12982859; doi:10.1177/20552076251410978)
Supplement: sj-docx-1-dhj-10.1177_20552076251410978 - Supplemental material for Attitudes toward artificial intelligence and its application in psychotherapy: Assessment in healthy adults and validation of an assessment measure [file sj-docx-1-dhj-10.1177_20552076251410978.docx]

Table S1. Group differences regarding the AI-scales separately presented for gender, education and therapy experience

| Group |  | *N* | ATAI_F | ATAI_A | ATUAIP |
| --- | --- | --- | --- | --- | --- |
| Gender | Men | 84 | 2.69 | 3.33 | 3.05 |
|  | Women | 116 | 3.00 | 2.96 | 2.67 |
|  | Difference (*W*) |  | 5944.5 | 3450 | 3598.5 |
|  | *p* |  | < 0.01 | < 0.01 | < 0.01 |
|  | *r* |  | 0.19 | -0.26 | -0.22 |
| Education | High educated | 83 | 2.65 | 3.18 | 2.96 |
|  | Low educated | 122 | 3.02 | 3.06 | 2.75 |
|  | Difference (*W*) |  | 6333 | 4577.5 | 4474.5 |
|  | *p* |  | < 0.01 | 0.23 | 0.16 |
|  | *r* |  | 0.22 | -0.08 | -0.10 |
| Therapy  experience | Psychotherapy experience | 77 | 2.96 | 2.99 | 2.74 |
|  | No experience | 128 | 2.82 | 3.18 | 2.88 |
|  | Difference |  | *W*=5348.5 | *W*=4186.5 | *T*=-1.1056 |
|  | *p* |  | 0.30 | 0.06 | 0.27 |
|  | *effectsize* |  | *r*=0.07 | *r*=-0.13 | *d*=-0.16 |

Notes. ATAI= Attitude Towards Artificial Intelligence; ATAI_A= Acceptance of Artificial Intelligence; ATAI_F= Fear of Artificial Intelligence; ATUAIP= Attitude toward the use of Artificial Intelligence in Psychotherapy. The value range of the respective variables of the scales is between 1 (“strongly disagree") and 5 ("strongly agree"), 3 (“neither nor”).

Table S2. Group differences regarding the AI-scales by symptom burden in the ISR-scales.

| Scale | Symptom  burden | Difference | *N* | ATAI_F | ATAI_A | ATUAIP |
| --- | --- | --- | --- | --- | --- | --- |
| Depression | No |  | 105 | 2.75 | 3.15 | 2.93 |
|  | Yes |  | 100 | 2.99 | 3.07 | 2.71 |
|  |  | (*W*) |  | 4181 | 5672 | 6064.5 |
|  |  | *p* |  | 0.011 | 0.308 | 0.055 |
|  |  | *r* |  | -.018 | 0.07 | -0.13 |
| Anxiety | No |  | 117 | 2.78 | 3.17 | 2.83 |
|  | Yes |  | 88 | 2.98 | 3.03 | 2.81 |
|  |  | (*W*) |  | 4296.5 | 5839 | 5218.5 |
|  |  | *p* |  | 0.041 | 0.092 | 0.867 |
|  |  | *r* |  | -0.14 | 0.12 | 0.01 |
| Compulsive-  obsessive | No |  | 124 | 2.73 | 3.19 | 2.80 |
|  | Yes |  | 81 | 3.09 | 2.99 | 2.86 |
|  |  | (*W*) |  | 3724.5 | 5718 | 4739 |
|  |  | *p* |  | 0.002 | 0.086 | 0.495 |
|  |  | *r* |  | -0.22 | -0.12 | -0.05 |
| Somatoform | No |  | 161 | 2.8 | 3.14 | 2.83 |
|  | Yes |  | 44 | 3.11 | 3.01 | 2.78 |
|  |  | (*W*) |  | 2802 | 3926.5 | 3587.5 |
|  |  | *P* |  | 0.032 | 0.258 | 0.897 |
|  |  | *r* |  | -0.15 | -0.08 | 0.01 |
| Eating | No |  | 138 | 2.82 | 3.08 | 2.79 |
|  | Yes |  | 67 | 2.97 | 3.18 | 2.89 |
|  |  | (*W*) |  | 4211 | 4399.5 | 4279.5 |
|  |  | *p* |  | 0.297 | 0.566 | 0.389 |
|  |  | *r* |  | -0.07 | -0.04 | -0.06 |
| Total scale | No |  | 116 | 2.76 | 3.18 | 2.86 |
|  | Yes |  | 89 | 3.01 | 3.02 | 2.78 |
|  |  | (*W*) |  | 4173.5 | 5954.5 | 5423 |
|  |  | *p* |  | 0.018 | 0.054 | 0.536 |
|  |  | *r* |  | -0.17 | 0.14 | - |

### Notes*.* ATAI= Attitude Towards Artificial Intelligence; ATAI_A= Acceptance of Artificial Intelligence; ATAI_F= Fear of Artificial Intelligence; ATUAIP= Attitude toward the use of Artificial Intelligence in Psychotherapy. The value range of the respective variables of the scales is between 1 (“strongly disagree") and 5 ("strongly agree"), 3 (“neither nor”). Classification of scores was conducted according to the cut-off values provided in the ISR manual.

Table S3. Differences in application areas.

| Application Area | Deskriptives | | Differences | | |  |
| --- | --- | --- | --- | --- | --- | --- |
|  | Mean | *SD* |  | *V* | *p* | r |
| Chatbots | 2.72 | 1.20 | To Robots | 5481 | <0.001 | 0.433 |
| Robots: | 2.29 | 1.19 | To App-based interventions | 802 | <0.001 | -0.739 |
| App-based interventions | 3.30 | 1.18 | To Chatbots | 5255 | <0.001 | 0.588 |
| Diagnostic | 3.23 | 1.20 | To Robots | 8732 | <0.001 | 0.727 |
| General use | 2.8 | 1.173 | To diagnostic | 2075 | <0.001 | -0.414 |

Table S4. Bivariate correlations of the AI-scales with the BFI and age

|  | A | E | O | N | C | ATAI_F | ATAI_A | ATUAIP | Age |
| --- | --- | --- | --- | --- | --- | --- | --- | --- | --- |
| Agreeableness (A) | 1 |  |  |  |  |  |  |  |  |
| Extraversion (E) | .13 | 1 |  |  |  |  |  |  |  |
| Openness (O) | .06 | .**24*** | 1 |  |  |  |  |  |  |
| Neuroticism (N) | -.05 | -.12 | .1 | 1 |  |  |  |  |  |
| Conscientiousness (C) | .18 | -.01 | .08 | -.12 | 1 |  |  |  |  |
| ATAI_F | -.06 | -.02 | .04 | .08 | .06 | 1 |  |  |  |
| ATAI_A | .04 | **-.16*** | -.08 | -.07 | **-.10*** | **-.52*** | 1 |  |  |
| ATUAIP | -.07 | -.02 | -.11 | - | -.07 | **-.38*** | **.45*** | 1 |  |
| Age | -.02 | -.1 | -.06 | -.16 | **.3*** | -.11 | - | -.07 | 1 |

### Note. *p* < 0.05. Notes. ATAI= Attitude Towards Artificial Intelligence; ATAI_A= Acceptance of Artificial Intelligence; ATAI_F= Fear of Artificial Intelligence; ATUAIP= Attitude toward the use of Artificial Intelligence in Psychotherapy.
